# A Highly Selective and Sensitive Fluorescent Turn-Off Probe for Cu^2+^ Based on a Guanidine Derivative

**DOI:** 10.3390/molecules22101741

**Published:** 2017-10-16

**Authors:** Fei Ye, Qiong Chai, Xiao-Min Liang, Ming-Qiang Li, Zhi-Qiang Wang, Ying Fu

**Affiliations:** Department of Applied Chemistry, College of Science, Northeast Agricultural University, Harbin 150030, China; yefei@neau.edu.cn (F.Y.); chaiqiong01@163.com (Q.C.); abcliangxiaomin@163.com (X.-M.L.); mqli@neau.edu.cn (M.-Q.L.); wzq19910101@163.com (Z.-Q.W.)

**Keywords:** fluorescence, naphthalimide, guanidine, octatomic ring, copper ion

## Abstract

A new highly selective and sensitive fluorescent probe for Cu^2+^, *N*-*n*-butyl-4-(1′-cyclooctene-1′,3′,6′-triazole)-1,8-naphthalimide (**L**), was synthesized and evaluated. The structure of compound **L** was characterized via IR, ^1^H-NMR, ^13^C-NMR and HRMS. The fluorescent probe was quenched by Cu^2+^ with a 1:1 binding ratio and behaved as a “turn-off” sensor. An efficient and sensitive spectrofluorometric method was developed for detecting and estimating trace levels of Cu^2+^ in EtOH/H_2_O. The ligand exhibited excitation and emission maxima at 447 and 518 nm, respectively. The equilibrium binding constant of the ligand with Cu^2+^ was 1.57 × 10^4^ M^−1^, as calculated using the Stern-Volmer equation. Ligand **L** is stable and can be used to detect Cu^2+^ in the range of pH from 7 to 12. The sensor responded to Cu^2+^ rapidly and a large number of coexisting ions showed almost no obvious interference with the detection.

## 1. Introduction

Fluorescent sensors for chemical species used in biological and environmental detection are currently an attractive field of research [1,2,3]. In particular, pollution by heavy metal ions has attracted much attention because these metal ions not only play important roles in various physiological processes but also have distinctly toxic impact on human health [4]. It is highly desired to detect these in relevant systems. The importance of metal ion sensing can never be overestimated because of their widespread distribution in environmental systems and biological processes [5]. Copper is a significant metal pollutant because of its widespread application and is also an essential trace element in biological systems. Cu^2+^ is present in various biological processes, as the third most abundant transition metal ion in the human body. In addition, its homeostasis is critical for the metabolism and development of living organisms [6,7,8]. For example, Cu^2+^ plays a critical role as a catalytic cofactor for a variety of metalloenzymes, including superoxide dismutase, cytochrome oxidase and tyrosinase [9,10]. The misregulation or excess accumulation of Cu^2+^ could cause serious diseases [11]. Its concentration in the neuronal cytoplasm may contribute to the etiology of Alzheimer’s or Parkinson’s disease [12,13,14]. However, exposure to high levels of Cu^2+^ will cause gastrointestinal disturbance and even liver or kidney damage. The detection of Cu^2+^ is meaningful for both disease diagnosis and environmental monitoring [15]. The normal average concentration of Cu^2+^ in blood is in the range of 15.7–23.6 μM [16]. The U.S. Environmental Protection Agency (EPA) set a limit for Cu^2+^ in drinking water of 20 μM [17]. The detection of Cu^2+^ levels in water requires an efficient, selective and highly sensitive chemosensor [18,19].

Numerous methods to detect metal ions have been developed, such as atomic absorption spectrometry [20], inductively coupled plasma atomic emission spectroscopy [21], visual detection [22,23], chemiluminescence detection [24] and electrochemical techniques [25,26]. However, these methods have disadvantages, such as expensive instrumentation, complicated sample preparation and tedious operating procedures, resulting in the need for other sensitive, simple detection methods for copper ions. Fluorescent molecules offer many advantages over other systems in this area as a result of their emissive behavior, easy signal detection, sensitivity, selectivity and low cost [27,28]. The development of the fluorescence method has been demonstrated to be promising because of its advantages of wide applicability, ease of manipulation, high selectivity, sensitivity, rapidity, nondestructive methodology and direct visual perception [29,30]. Thus, a number of high-performance sensors have been developed to detect metal ions [31]. Cu^2+^ is a typical ion with a pronounced quenching effect on fluorophores via mechanisms that are inherent to the paramagnetic species [32,33,34,35].

**Scheme 1:**
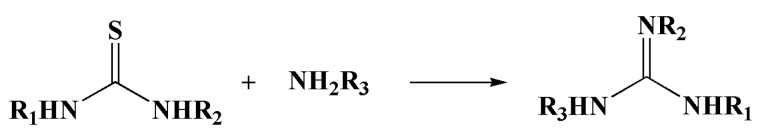
Thiourea transformation into guanidine.

Many excellent chemosensors have been reported for Cu^2+^ in the past few years. However, many of these sensors only exhibit sensitive and selective Cu^2+^ detection in pure organic systems (Table 1) [36,37,38]. Hence, we designed and synthesized a new fluorescent sensor, *N*-*n*-butyl-4-(1′-cyclooctene-1′,3′,6′-triazole)-1,8-naphthalimide, based on the transformation of thiourea into guanidine derivatives, as shown in Scheme 1. This novel compound has a highly sensitive fluorescent response toward Cu^2+^ in mixed aqueous solutions.

## 2. Results and Discussion

### 2.1. Synthesis and Characterization

**Scheme 2:**
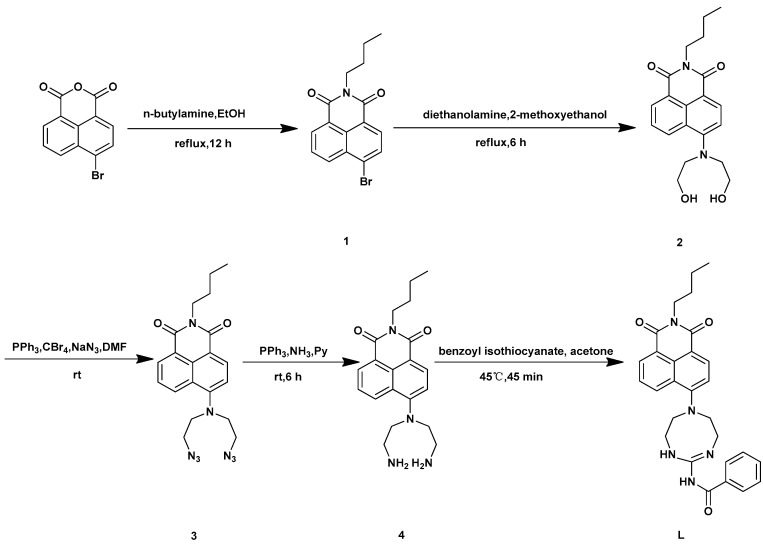
Synthetic route to compound **L**.

Compound **L** was synthesized through the route outlined in Scheme 2. The structure modification was focused on the C-4 position. A dihydroxyethylamino moiety was introduced to replace the Br atom, and the subsequent reaction modified the -OH group to N_3_; compound **4** was obtained in a 67% yield from the amination. Thiourea is easily transformed into guanidine; however, the conversion from thiourea to guanidine has attracted little attention as a potential fluorogenic reaction because most thiourea derivatives have the same color or fluorescence as their guanidine analogues [39]. Initially, we envisioned that a similar molecular recognition/reactivity motif might be incorporated into fluorophores to serve as a ratiometric fluorescent chemodosimeter for the selective detection of Hg^2+^, as reported in the literature [40], because compound **4** has two free amino groups. The expected thiourea derivative was not achieved; however, the product transformed into the inner guanidine ring because compound **4** contained a primary amine subunit. Compound **L** was obtained through intramolecular cyclization with a recognition/reactivity motif that could be incorporated into fluorophores so that metal ion addition led to a variation of the fluorescence.

Compound **L** was obtained with a yield of 57% in the final reaction, and its structure was characterized by ^1^H-NMR, ^13^C-NMR and HRMS. As evidenced by the signals at 6.57–8.42 ppm in the ^1^H-NMR spectrum, the number of Ar-H is 10, which indicated that benzoyl isothiocyanate had reacted with compound **4**. The signals at δ = 9.02 ppm indicated the existence of benzamide, and the signals at δ = 1.26 ppm showed that -NH_2_ was substituted. Furthermore, the molecular ion peak in the HRMS-ESI ([M + H]^+^, 484.2332 *m*/*z*) spectrum was consistent with the theoretically calculated molecular mass of compound **L**.

### 2.2. Spectral Characteristics

**Figure 1:**
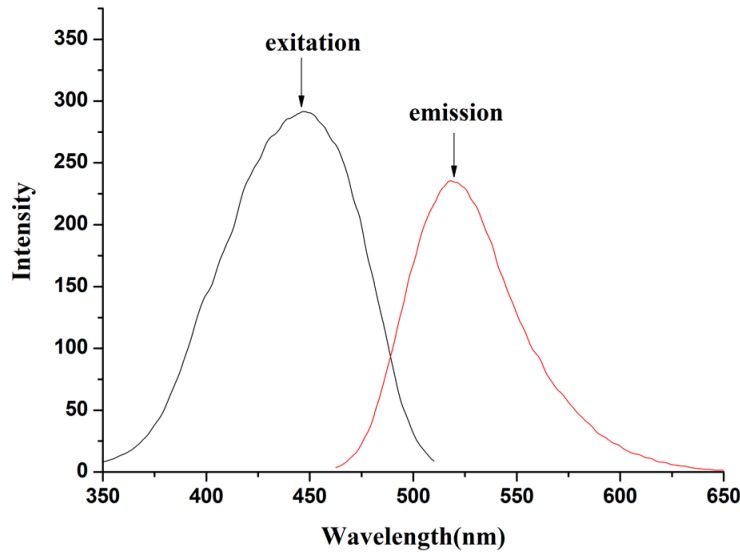
Excitation and emission spectra of compound **L** (10 μM) in EtOH/H_2_O (4:1 *v*/*v*).

The photophysical characteristics of compound **L** in EtOH/H_2_O (4:1 *v*/*v*) were investigated. Figure 1 shows the excitation (*λ* = 447 nm) and emission (*λ* = 518 nm) spectra of compound **L**. The Stokes shift (*υ_A_*–*υ_F_*), an important parameter indicating the difference in properties and structure between the ground state (*S*_0_) and first excited state (*S*_1_), was calculated by Equation (1) to be 3062 cm^−1^.

(1) $$(\upsilon_{A}-\upsilon_{F})=(1/\lambda_{A}-1/\lambda_{F})\times 10^{-7}{\mathrm{cm}}^{-1}$$

The ability of the molecules to emit absorbed light energy is characterized quantitatively by the fluorescence quantum yield (Φ_F_). The quantum yields of fluorescence were calculated using coumarin 6G (Φ_sample_ = 0.94) in EtOH as the standard according to Equation (2), where *A_ref_*,* S_ref_*, and *n_ref_* and *A_sample_*, *S_sample_*, and *n_sample_* represent the absorbance at the exited wavelength, the integrated emission band area and the solvent refractive index of the standard and the sample, respectively [41,42]. Thus, the fluorescence quantum yield of probe **L** in EtOH/H_2_O (4:1 *v*/*v*) solution was 0.06.

(2) $$\Phi_{F}=\Phi_{\mathrm{ref}}\left(\frac{S_{sample}}{S_{ref}}\right)\left(\frac{A_{ref}}{A_{sample}}\right)\left(\frac{{n^{2}}_{sample}}{{n^{2}}_{ref}}\right)$$

The photophysical properties of compound **L** as a ligand in the presence of different metal cations were investigated in EtOH/H_2_O (4:1 *v*/*v*) to determine its potential application as a sensor for detecting these metal ions. The binding behavior of compound **L** toward different metal ions such as their corresponding chlorides, acetates, sulfides or nitrates was studied using fluorescence spectroscopy. The anions did not affect the spectroscopic results.

**Figure 2:**
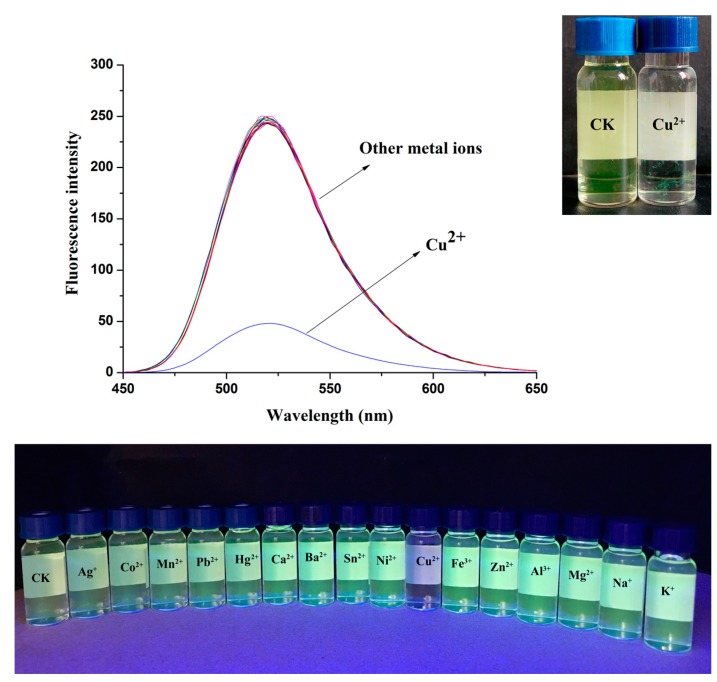
Fluorescence changes of compound **L** (10 μM) in the absence (CK) and presence of metal cations (50 μM) in EtOH/H_2_O (4:1 *v*/*v*).

To evaluate the selectively of compound **L** for Cu^2+^, changes in the fluorescence intensity upon the addition of various metal ions under the same conditions were investigated. The fluorescence spectrum of compound **L** exhibited strong fluorescence emission when excited at 447 nm in EtOH/H_2_O (4:1 *v*/*v*; Figure 2). Under the same conditions as those used for adding the metal ions, significant fluorescence changes were observed in the presence of Cu^2+^. Fluorescence quenching was observed immediately after Cu^2+^ was added. When other metal ions were added, the fluorescence exhibit few changes. As shown in Figure 2, remarkable color changes ranging from yellow to colorless and yellow floccules floating in the solution could be distinguished by the naked eye. In addition, the introduction of Cu^2+^ to the mixture solution of compound **L** led to evident fluorescence quenching, while the rest of the metal cations exerted a negligible or very small influence under identical conditions, which was further evidenced by the illumination taken under a 365 nm portable UV lamp. On the basis of the literature, the fluorescence quench of probe **L** might have been due to the coordination to paramagnetic Cu^2+^ [43].

### 2.3. Calculation of Binding Constants and Different Concentrations of Cu^2+^

**Figure 3:**
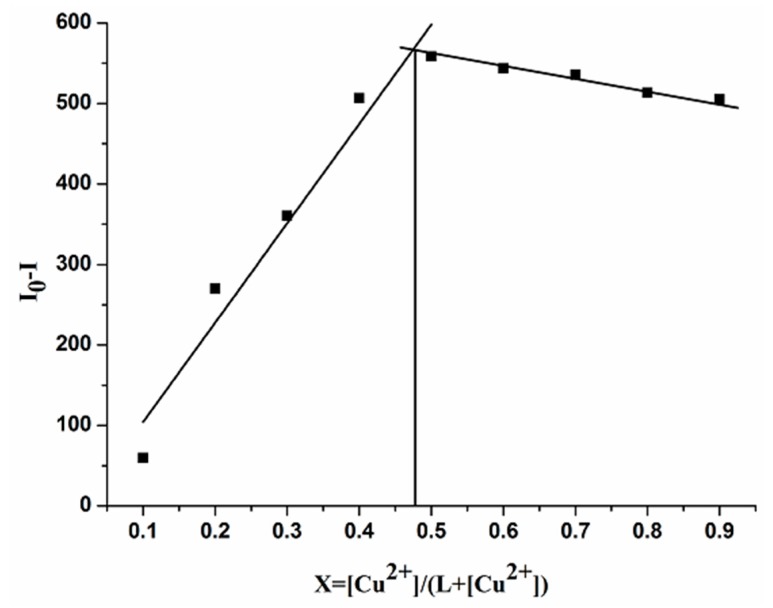
Job plot to determine the stoichiometry of the [**L**-Cu^2+^] complex in solution.

A Job plot was generated by continuously varying the mole fraction of Cu^2+^ from 0 to 1 in a solution of [**L**-Cu^2+^] with a total concentration of 50 μM to determine the stoichiometry of the [**L**-Cu^2+^] complex. The Job plot analysis revealed an approximate maximum at a 0.5 mole fraction, which indicated a 1:1 stoichiometry of the ligand **L** and Cu^2+^ complex (Figure 3).

**Figure 4:**
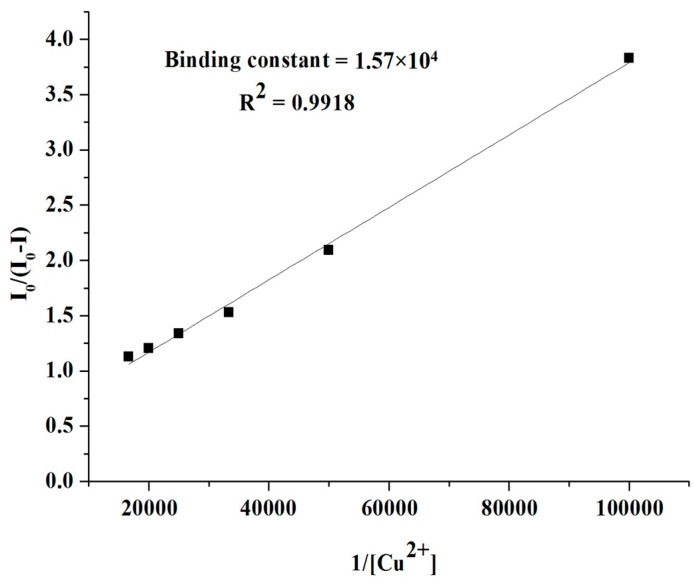
Stern-Volmer plot for calculating the binding constant of the chemosensor with Cu^2+^.

To better understand the sensing process, changes in the fluorescence spectra of compound **L** were measured along with the binding constant and different concentrations of Cu^2+^. The binding constant was calculated using the Stern-Volmer equation, *I*_0_/*(I*_0_ − *I)* = 1/*A* + 1/*KA*·1/[*Q*], on the basis of a 1:1 stoichiometry, where *I*_0_ is the fluorescence intensity of the free ligand **L**, *I* is the fluorescence intensity of the **L**-Cu^2+^ complex, *Q* is [Cu^2+^], *A* is a constant and *K* is a binding constant [44]. When the reciprocal of *I*_0_/*(I*_0_ − *I)* was plotted as a function of the 1/[*Q*] concentration, a linear relationship was obtained, (*y* = A + B*x*), *y* = *I*_0_/*(I*_0_ − *I)*, A = 1/*A*, B = 1/*KA*, *x =* 1/[*Q*], and *K* was calculated from A/B (Figure 4). Therefore, *K*, the binding association constant for Cu^2+^, was estimated to be 1.57 × 10^4^ M^−1^ in the EtOH/H_2_O (4:1 *v/v*) solution, as inferred from the fluorescence titration curves of compound **L** with Cu^2+^.

**Figure 5:**
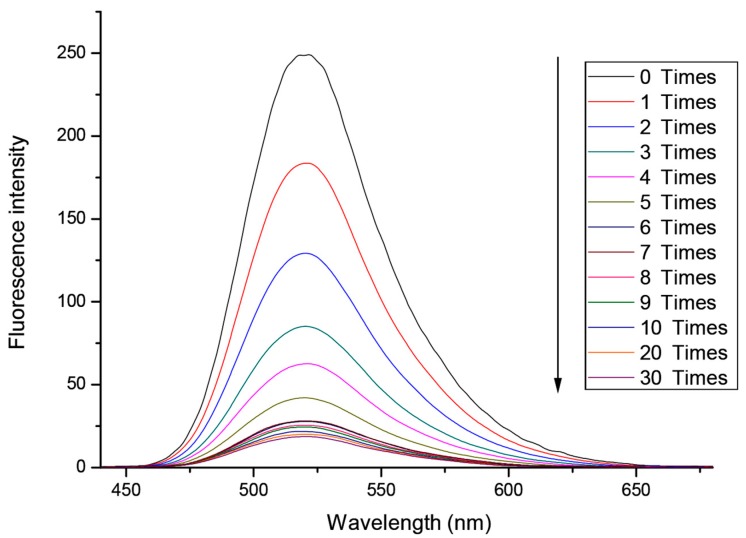
Changes in the fluorescence spectra of compound **L** (10 μM) in EtOH/H_2_O (4:1 *v/v*) as a function of added [Cu^2+^].

**Figure 6:**
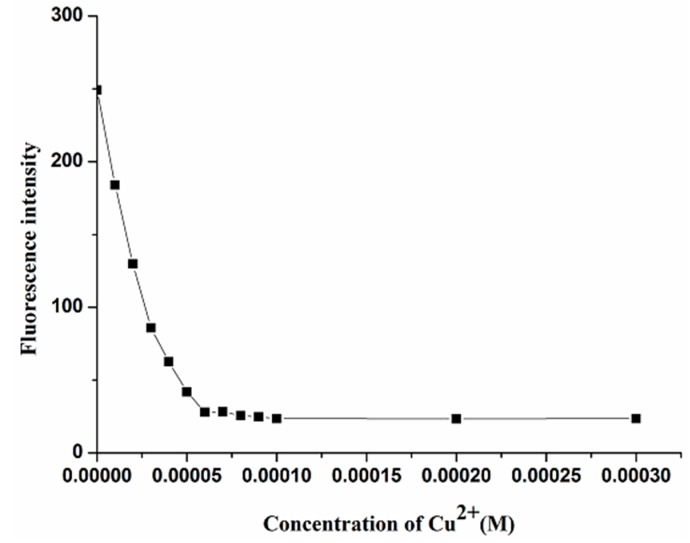
Fluorescence intensity vs Cu^2+^; inset shows that up to 6-fold Cu^2+^ curvilinearity was sustained; *λ*_ex_: 437 nm; *λ*_em_: 518 nm; slit width: 10 nm.

Upon the addition of Cu^2+^, the fluorescence was quenched. The changes in the fluorescence emission intensities of compound **L** (1 × 10^−5^ mol L^−1^) with different concentrations of Cu^2+^ (0–3 × 10^−4^ mol L^−1^) are presented in Figure 5. A progressive decrease in the fluorescence intensity was observed. The plot of the fluorescence intensity versus externally added Cu^2+^ (Figure 6) reaches saturation after a certain amount of externally added Cu^2+^. Curvilinearity was obtained for up to a value of 6 times (6 × 10^−5^ mol L^−1^) the amount of externally added Cu^2+^. The results indicated that compound **L** could detect Cu^2+^ at a micromole level.

### 2.4. Response Time

**Figure 7:**
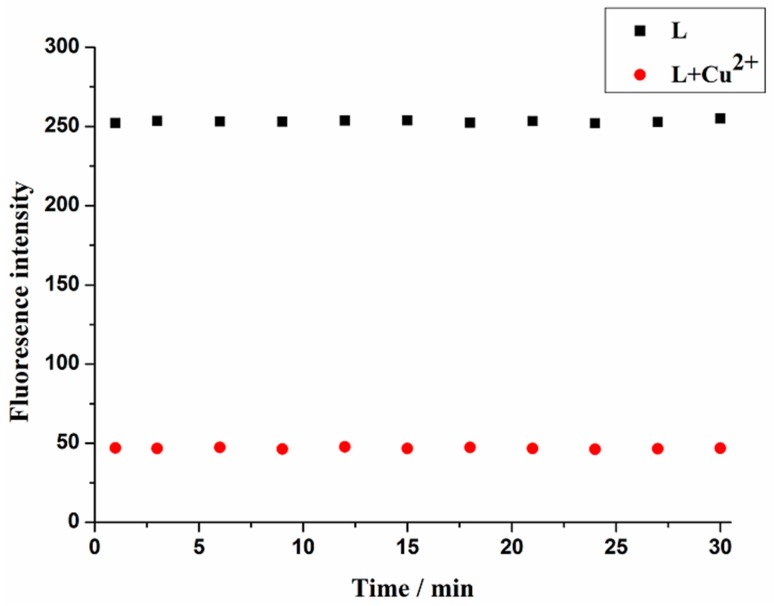
Response time of 10 μM probe **L** in the absence or presence of 50 μM Cu^2+^ in EtOH/H_2_O (4:1 *v/v*); *λ*_ex_: 437 nm; *λ*_em_: 518 nm; slit width: 10 nm.

The response time is a significant practical parameter [45]. Time-dependence of probe **L** for sensing Cu^2+^ was examined and the results are illustrated in Figure 7. Upon the addition of Cu^2+^, the fluorescence intensity decreased to the minimum immediately and tended to be stable during the test time. No changes in the fluorescence of probe **L** in the absence of Cu^2+^ were detected with the increased time. This showed that probe **L** exhibited a reliable rapid response to Cu^2+^.

### 2.5. Effect of pH

**Figure 8:**
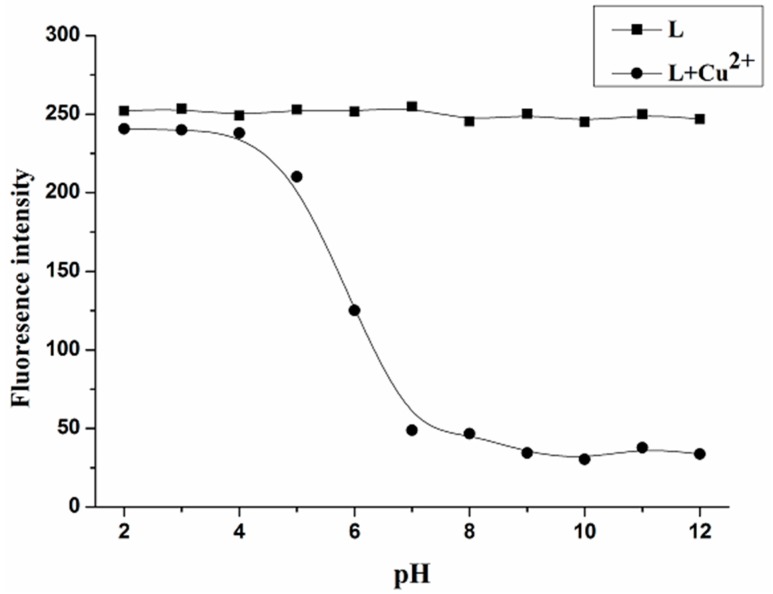
Effect of pH on the fluorescence intensity of 10 μM probe **L** in the absence or presence of 50 μM Cu^2+^.

In order to investigate the application of probe **L** within complex media, the fluorescence spectra response of probe **L** was measured in the absence or presence of 5 × 10^−5^ mol L^−1^ Cu^2+^ under different pH conditions. As shown in Figure 8, the fluorescence intensity of probe **L** did not reveal any clear differences over a pH range of 2–12, which was consistent with the goal of our experimental design. The fluorescence of the [**L**-Cu^2+^] solution could not remarkably decrease when the pH values were lower than 5, possibly because the imino groups of probe **L** were protonated at acid conditions, resulting in weak coordination with Cu^2+^. In addition, the fluorescence intensity of the [**L**-Cu^2+^] solution showed a 5-fold change from pH 4 to pH 7, which suggested that it is sensitive to pH changes. These results indicated that probe **L** could be used to detect biological as well environmental Cu^2+^ in the range of pH from 7 to 12.

### 2.6. Selectivity

**Figure 9:**
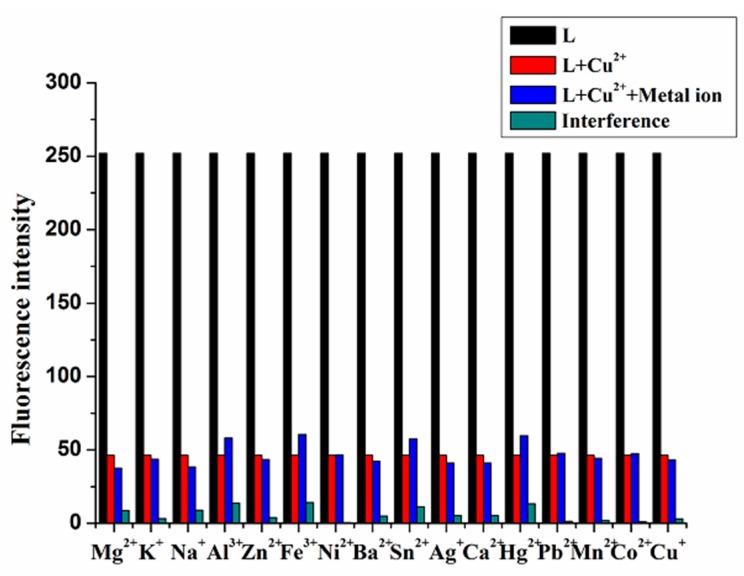
Interference of foreign cations. Solvents: EtOH/H_2_O (4:1 *v/v*).

The selectivity is clearly one of the most important characteristics of a cation-selective chemosensor. Thus, the influence of other metal ions on the fluorescence intensity of the proposed Cu^2+^ chemosensor was investigated (Figure 9). In this study, Ag^+^, Na^+^, K^+^, Ba^2+^, Ca^2+^, Co^2+^, Mg^2+^, Mn^2+^, Ni^2+^, Pb^2+^, Sn^2+^, Zn^2+^, Al^3+^, Cu^+^, Hg^2+^ and Fe^3+^ (100 μM) were added to the compound **L** (10 μM)/Cu^2+^ (50 μM) solution, and the fluorescence was observed. The fluorescence emission intensity at 518 nm significantly decreased as a result of binding with Cu^2+^. The coexisting ions showed little influence on the detection of Cu^2+^. Common anions, including oxalate, sulfate, nitrate and chloride, did not interfere with the fluorescence intensity of the ligand or [**L**-Cu^2+^] complex. Compared to the other common cations, compound **L** exhibited good selectivity for Cu^2+^, indicating that **L** has a good anti-interference ability in detecting Cu^2+^.

### 2.7. Thermal Studies

**Figure 10:**
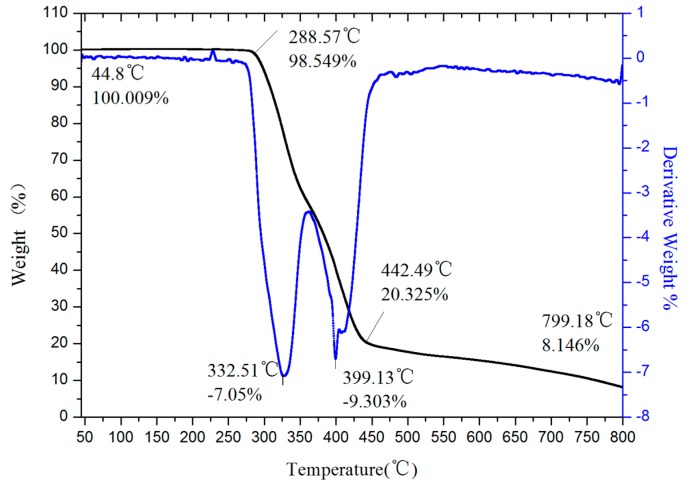
Thermal studies of the fluorescence chemosensor.

**Figure 11:**
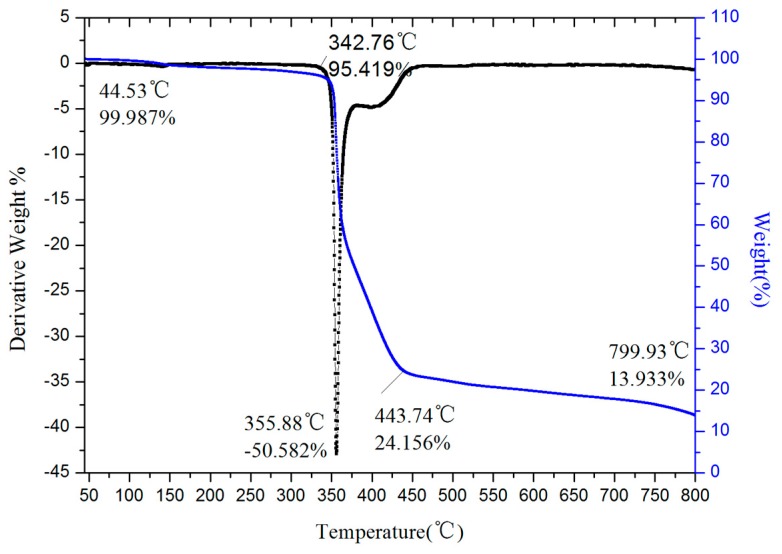
Thermal studies of the [**L**-Cu^2+^] complex with the fluorescent chemosensor.

The stability of probe **L** and its Cu^2+^ complex was studied by thermogravimetry to prove the binding of ligand **L** with Cu^2+^ ions, and the results are presented in Figure 10 and Figure 11, respectively. The thermal stability of the Cu^2+^ complex (up to 324.76 °C) was better than that of the free ligand (up to 288.57 °C).

### 2.8. IR Spectra

**Figure 12:**
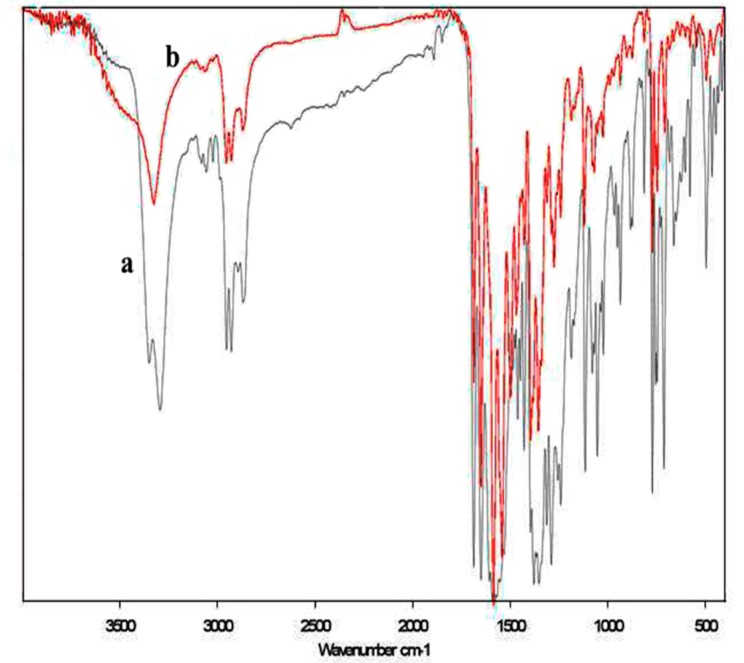
IR spectra of compound **L** before (a) and after (b) the addition of Cu^2+^.

**Scheme 3:**
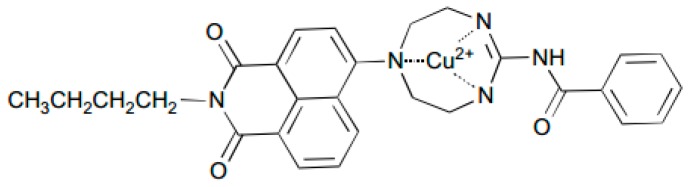
Proposed coordination of compound **L** combined with Cu^2+^.

The structural matching played an important role in selective recognition. According to the examples in the literature, a similar mode of probes containing a tridentate structure binding with Cu^2+^ has been reported [46,47]. To further study the possible binding mode of compound **L** and Cu^2+^, IR spectroscopy was employed to elucidate the possible coordination mode. The IR spectra of compound **L** and the [**L**-Cu^2+^] complex are compared in Figure 12. The IR spectra of compound **L** (Figure 12a) has two slender peaks appearing between 3350 and 3290 cm^−1^. One of the extremely sharp peaks in this range vanished when the complex was formed (Figure 12b) and only appeared at 3325.72 cm^−1^. This result suggested that the aromatic amide was not involved in the coordination of [**L**-Cu^2+^]. However, the extremely sharp peak in the IR spectra corresponding to the imine disappeared. For fluorescence quenching, the N atom directly connected to naphthalene must be present in the coordination of the complex. On the basis of the aforementioned fact, a plausible binding mode of the complex was proposed, as shown in Scheme 3.

## 3. Experimental

### 3.1. Chemicals and Instruments

All of the solvents and reactants were commercially available and used without purification. The IR spectra were recorded on a Bruker ALPHA-T spectrometer (KBr, BRUKER Inc., Beijing, China). The melting points were determined using a Beijing Taike melting point apparatus (X-4) and were uncorrected, and ^1^H-NMR and ^13^C-NMR spectra were recorded on a Bruker AVANVE 300 or 400 MHz nuclear magnetic resonance spectrometer (Energy Chemical, Shanghai, China) using CDCl_3_ or DMSO-*d*_6_ as the solvent and TMS as the internal standard. The high-resolution mass spectrometry was recorded on a FT-ICR MS spectrometer (BRUKER Inc., Beijing, China). Fluorescence emission spectra were obtained using a PerkinElmer LS55 fluorescence spectrometer (PerkinElmer, Liantrisant, UK). The pH values were measured using a PHS-3C pH meter (Inesa, Shanghai, China). Thermal analysis was performed using a 449F3 thermal analyzer (Netzsch, Bavaria, Germany). The spectrogram data of the synthesized compounds could be found in the Supplementary Materials.

### 3.2. Testing Methods

Compound **L** was dissolved in EtOH to form a 10^−3^ M stock solution. Cu^2+^, Ag^+^, Na^+^, K^+^, Ba^2+^, Ca^2+^, Co^2+^, Mg^2+^, Mn^2+^, Ni^2+^, Pb^2+^, Sn^2+^, Zn^2+^, Al^3+^, Hg^2+^ and Fe^3+^ sources were dissolved in deionized water (H_2_O) to obtain 10^−2^ M stock solutions. For the fluorescence measurement of Cu^2+^, the mixed stock solutions were diluted to 10 mL with EtOH and H_2_O to form EtOH/H_2_O (4:1 *v/v*) solutions. For the titration experiments, compound **L** stock solution (100 μL) was mixed with a certain amount of Cu^2+^ stock solution and diluted to 10 mL with EtOH and H_2_O to form EtOH/H_2_O (4:1 *v/v*) solutions. The wide pH range solutions were prepared by adjustment of 0.05 mol L^−1^ Tris-HCl solution with HCl or NaOH solution. Thermal studies of compound **L** and its [**L**-Cu^2+^] complex were also performed to prove the binding of the ligand with Cu^2+^. All fluorescence spectra were recorded at 25 °C with the excitation wavelength set at 437 nm and the slit width set at 10 nm.

### 3.3. Synthesis of N-n-Butyl-4-bromo-1,8-naphthalimide *(**1**)*

*N*-*n*-Butyl-4-bromo-1,8-naphthalimide was prepared according to the reported method [48]. 4-Bromo-1,8-naphthalic anhydride (16.1 g, 58 mmol) and *n*-butyl amine (60 mmol, 4.4 g) were heated under reflux in EtOH (250 mL) with vigorous stirring for 12 h under N_2_. Then, the mixture was cooled, and the precipitate was filtered and recrystallized from ethanol to yield 13.5 g (84%) of a light-yellow product; m.p.: 105–106 °C; ^1^H-NMR (DMSO-*d*_6_, 300 MHz) δ (ppm): 8.46–8.52 (m, Ar-H, 2H), 8.27 (d, *J* = 7.9 Hz, Ar-H, 1H), 8.16 (d, *J* = 7.9 Hz, Ar-H, 1H), 7.92–7.97 (m, Ar-H, 1H), 4.01 (t, *J* = 7.3 Hz, N-CH_2_, 2H), 1.56–1.66 (m, CH_2_, 2H), 1.31–1.39 (m, CH_2_, 2H), and 0.93 (t, *J* = 7.3Hz, CH_3_, 3H).

### 3.4. Synthesis of N-n-Butyl-4-N′,N′-dihydroxyethyl-1,8-naphthalimide *(**2**)*

*N*-*n*-butyl-4-(*N*′,*N*′-dihydroxyethyl)amino-1,8-naphthalimide was obtained using a procedure similar to that reported by Guo et al. [49]. N-*n*-butyl-4-bromine-1,8-naphthalimide (15 g, 45.2 mmol) and diethanolamine (75 mL) were mixed in ethylene glycol monomethyl ether (100 mL). The mixture was refluxed for 6 h. The crude product was purified by column chromatography using silica gel, with EtOAc and light petroleum (1:4 *v/v*) as the eluent. Compound **2** was obtained as a yellow solid (3.28 g) in a 20% yield; m.p.: 129–130 °C; ^1^H-NMR (400 MHz, CDCl_3_) δ (ppm): 8.87 (d, *J* = 8.8 Hz, Ar-H, 1H), 8.52 (d, *J* = 7.2 Hz, Ar-H, 2H), 7.66 (t, *J* = 8.0 Hz, Ar-H, 1H), 7.38 (t, *J* = 8.0 Hz, Ar-H, 1H), 4.14 (t, *J* = 7.2 Hz, CH_2_, 2H), 3.87 (t, *J* = 5.2 Hz, CH_2_, 4H), 3.63 (t, *J* = 5.2 Hz, CH_2_, 4H), 2.81 (s, OH, 2H), 1.68–1.69 (m, CH_2_, 2H), 1.43–1.45 (m, CH_2_, 2H), 0.97 (t, *J* = 7.2 Hz, CH_3_, 3H); ^13^C-NMR (100 MHz, CDCl_3_) δ (ppm): 164.34, 163.91, 154.28, 131.18, 131.22, 130.85, 130.14, 127.24, 125.65, 122.92, 117.18, 117.05, 59.73, 59.73, 55.33, 55.33, 40.10, 30.20, 20.38, and 13.85.

### 3.5. Synthesis of N-n-Butyl-4-N′,N′-diaminoethyl-1,8-naphthalimide *(**4**)*

*N*-*n*-butyl-4-*N*′,*N*′-diaminoethyl-1,8-naphthalimide was prepared according to the reported procedure [50]. Compound **2** (2.0 g, 5.6 mmol), PPh_3_ (4.41 g, 16.8 mmol) and NaN_3_ (1.82 g, 28.0 mmol) were dissolved in anhydrous *N*,*N*-dimethylformamide (100 mL), the solution was cooled to 0 °C, and CBr_4_ (5.58 g, 16.8 mmol) was added portionwise over 10 min. Then, the reaction temperature was raised to room temperature, and the mixture was stirred for 12 h. The reaction mixture was diluted with water and extracted with ethyl acetate (4 × 50 mL). The organic layer was dried over anhydrous Na_2_SO_4_ and concentrated in vacuo. The crude compound was separated through silica gel column chromatography and afforded compound **3**. The compound **3** (1.9 g, 4.67 mmol) and PPh_3_ (7.36 g, 28.1 mmol) were dissolved in 50 mL of pyridine and kept at room temperature for 1 h. Concentrated ammonium hydroxide (10 mL) was added, and the mixture was allowed to stand for an additional 2 h. Pyridine was removed through reduced pressure. Then, CH_2_Cl_2_ and 1 N HCl were added to the reaction mixture. The organic layer was washed with additional 1 N HCl, and the aqueous layers were combined, neutralized with KOH, and extracted with CH_2_Cl_2_. The combined organic layers were washed with water and brine and then dried over anhydrous Na_2_SO_4_ to afford compound **4** (1.11 g, 67%) as a yellow solid; m.p.: 99–101 °C; ^1^H-NMR (DMSO-*d*_6_, 300 MHz) δ (ppm): 8.74 (d, *J =* 0.3 Hz, Ar-H, 1H), 8.43 (d, *J =* 7.8 Hz, Ar-H, 1H), 8.27 (d, *J =* 8.4 Hz, Ar-H, 1H), 7.68 (t, *J =* 8.1 Hz, Ar-H, 1H), 6.81 (d, *J =* 8.4 Hz, Ar-H, 1H), 4.01 (t, *J =* 7.8 Hz, CH_2_, 2H), 3.44–3.48 (m, CH_2_, 2H), 2.87 (t, *J =* 5.7 Hz, CH_2_, 2H), 2.65–2.72 (m, CH_2_, 4H), 1.84 (s, NH_2_, 4H), 1.54–1.62 (m, CH_2_, 2H), 1.30–1.37 (m, CH_2_, 2H), and 0.92 (t, *J =* 7.5 Hz, CH_3_, 3H). HRMS-ESI *m*/*z*: found 355.2123 for [M + H]^+^ and calculated 354.2056 for C_20_H_26_N_4_O_2_.

### 3.6. Synthesis of N-n-Butyl-4-(1′-cyclooctene-1′,3′,6′-triazole)-1,8-naphthalimide *(**L**)*

*N*-*n*-butyl-4-(1′-cyclooctene-1′,3′,6′-triazole)-1,8-naphthalimide was obtained using a procedure similar to that reported in the literature [40]. Benzoyl isothiocyanate (0.92 g, 5.6 mmol) was added dropwise to a solution of *N*-*n*-butyl-4-*N*′,*N*′-diaminoethyl-1,8-naphthalimide (1.0 g, 2.8 mmol) in 30 mL of ketone and was stirred at 45 °C. The reaction mixture was stirred continuously for 45 min. After cooling, the solution was filtered, and the filter cake was washed with EtOH. The crude product was purified by chromatography to afford compound **L** (0.77 g, 57%) as a green solid; m.p.: 165–167 °C; ^1^H-NMR (CDCl_3_, 300 MHz) δ (ppm): 9.02 (s, NH, 1H), 8.29–8.42 (m, Ar-H, 4H), 7.54 (d, *J =* 6.6 Hz, Ar-H, 1H), 7.46 (t, *J =* 7.5 Hz, Ar-H, 3H), 6.57 (d, *J =* 8.7 Hz, Ar-H, 2H), 4.12 (t, *J =* 7.5 Hz, CH_2_, 4H), 3.60–3.86 (m, CH_2_, 4H), 3.55–3.61 (m, CH_2_, 2H), 1.63–1.70 (m, CH_2_, 2H), 1.38–1.45 (m, CH_2_, 2H); 1.26 (s, CNHC, 1H), 0.95 (t, *J =* 7.5 Hz, CH_3_, 3H). ^13^C-NMR (75 MHz, CDCl_3_) δ (ppm): 164.72, 164.26, 164.26, 150.97, 150.97, 134.24, 134.24, 131.02, 131.02, 129.54, 129.54, 128.35, 128.35, 128.35, 128.35, 124.25, 122.59, 110.26, 103.13, 103.13, 45.86, 41.31, 41.24, 41.05, 39.93, 30.32, 20.44 and 13.88. HRMS-ESI *m*/*z*: found 484.2332 for [M + H]^+^ and calculated 483.5616 for C_28_H_29_N_5_O_3_.

## 4. Conclusions

In summary, a new, rapidly responsive, colorimetric and “turn-off” fluorescent Cu^2+^ sensor, *N*-*n*-butyl-4-(1′-cyclooctene-1′,3′,6′-triazole)-1,8-naphthalimide, was successfully synthesized and characterized. Probe **L** exhibited a markedly quenched fluorescence in the presence of Cu^2+^ with a simultaneous color change (yellow to colorless) over a range of metal cations. The detection was highly sensitive and more selective than some reported probes and almost unaffected by the common coexisting metal ions [15,51]. In addition, Probe **L** showed a 1:1 stoichiometry (sensor/Cu^2+^) with a high binding constant. This sensor should be valuable for copper analysis in environmental samples and biological systems.

## Figures and Tables

**Table 1:** Comparison of the recently reported sensors for the determination of Cu^2+^ ions.

Operation Mode	Solvent	*λ*_ex_/*λ*_em_ (nm)	Interference	References
Turn-off	CH_3_CN	364/420	None	[36]
Turn-on	CH_3_CN	365/475	Zn(II)	[37]
Turn-off	MeOH	480/518	None	[38]
Turn-off	EtOH/H_2_O	437/518	None	This work

## Supplementary Materials

Supplementary data associated with this article can be found in the online.
