# Supplementary material for: A Highly Selective and Sensitive Fluorescent Turn-Off Probe for Cu2+ Based on a Guanidine Derivative
Source: Molecules. 2017 Oct 16;22(10):1741. doi: 10.3390/molecules22101741 (PMC6151758; doi:10.3390/molecules22101741)

Compound 4

IR

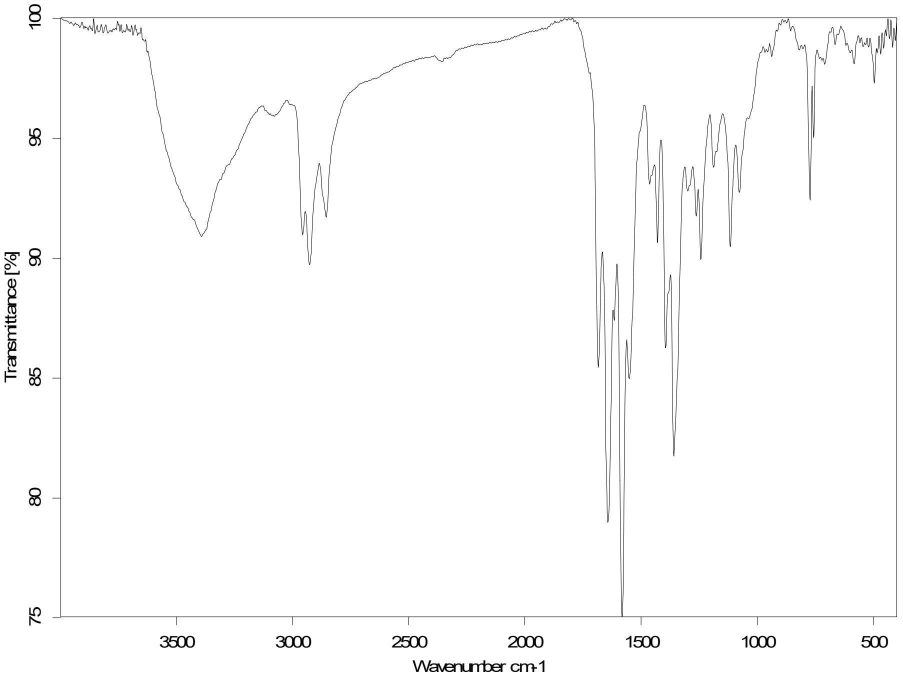

^{1}H NMR

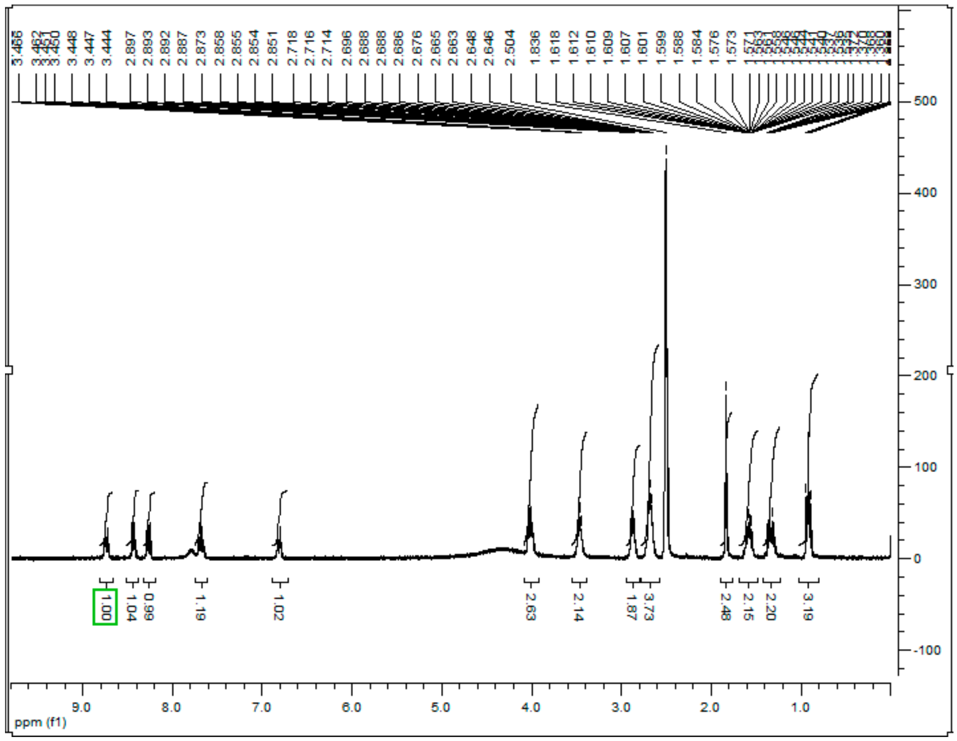

ESI-MS

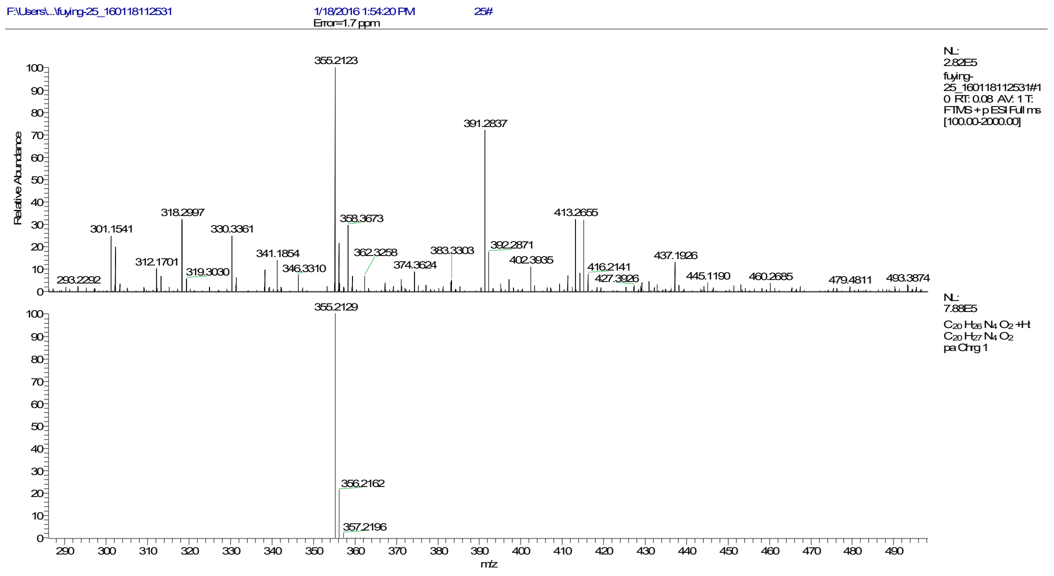

Compound L

IR

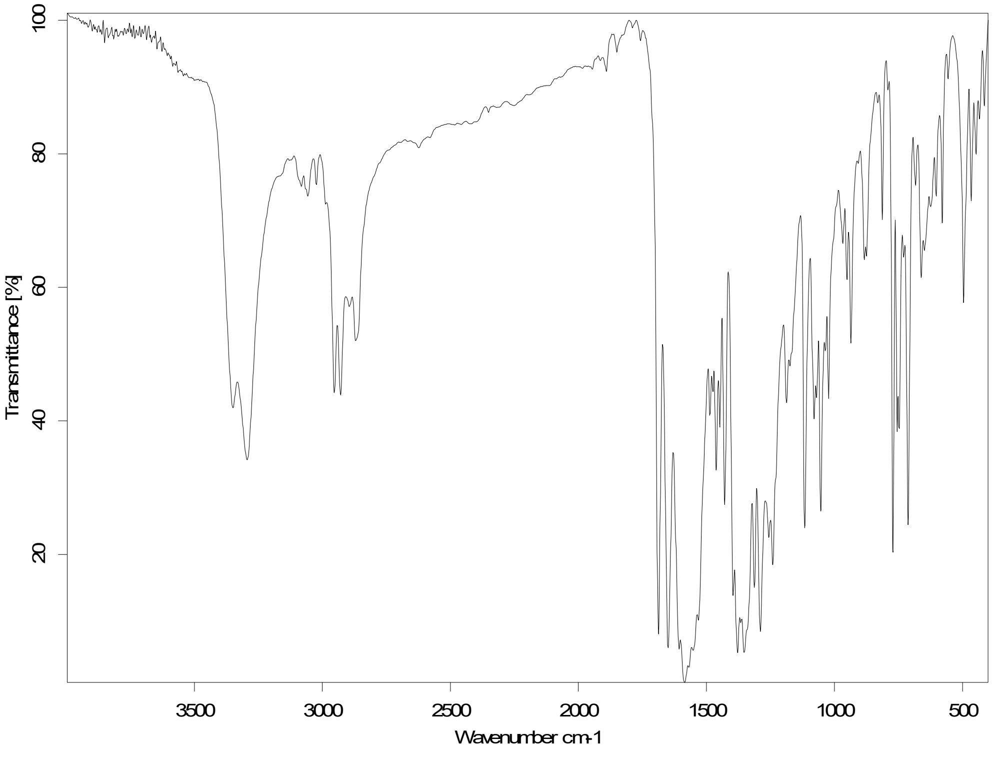

^{1}H NMR

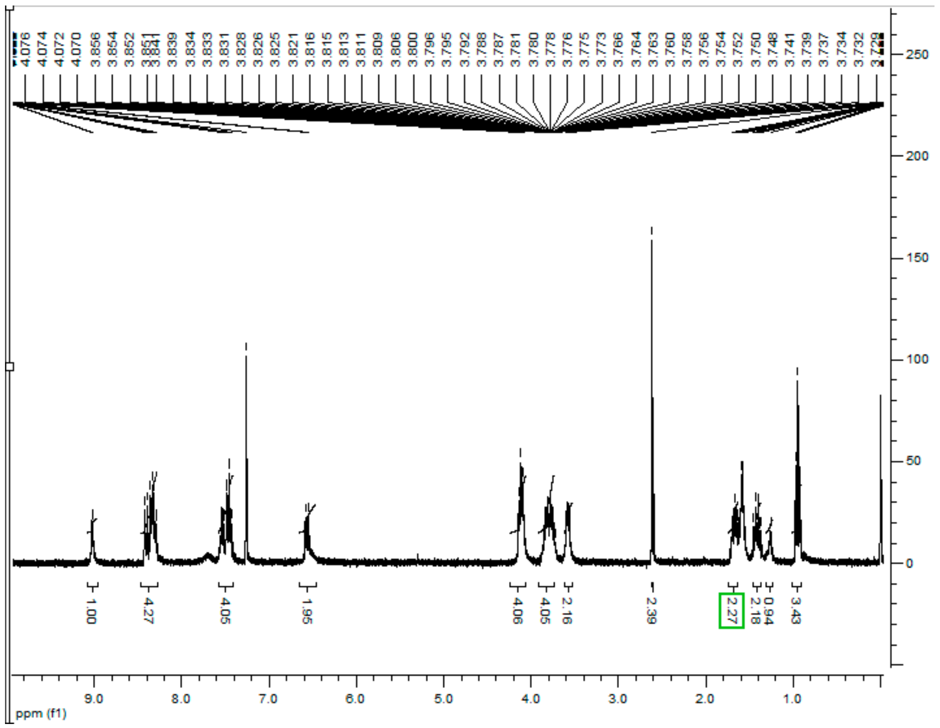

^{13}C NMR

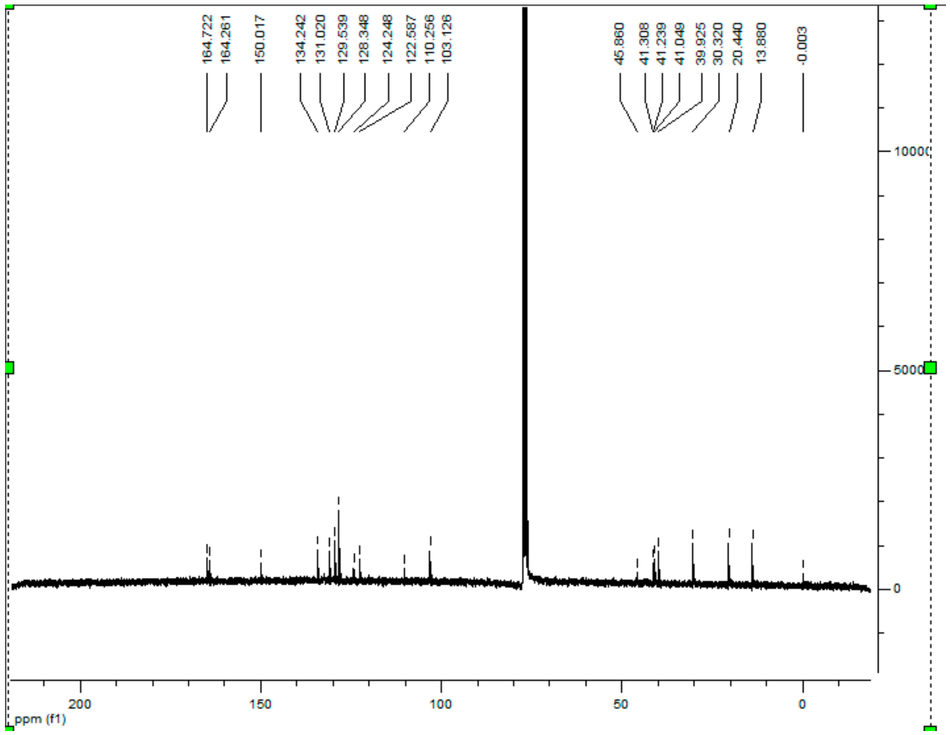

ESI-MS

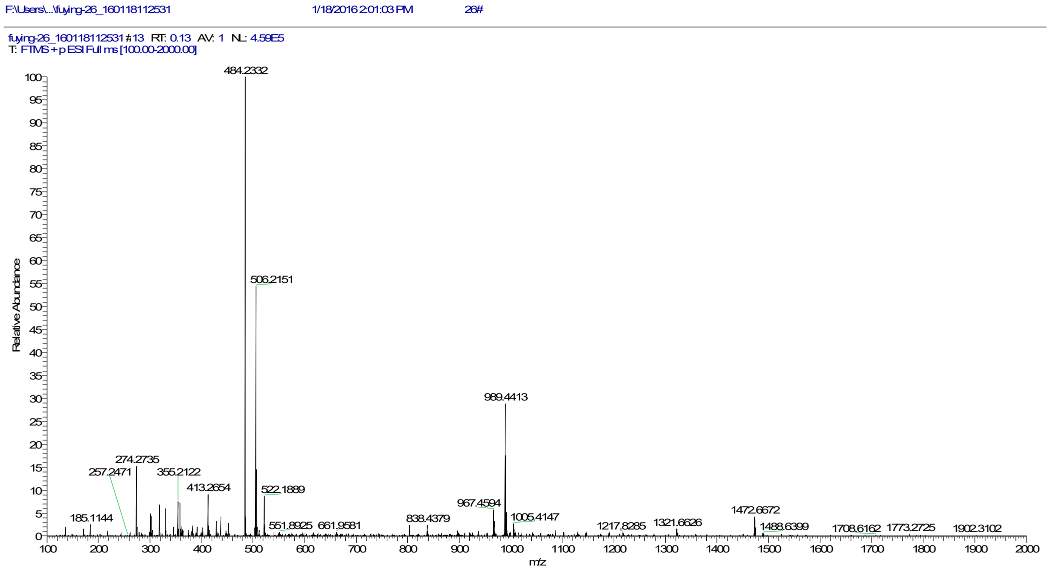

Cu (II)-**L** complex

IR

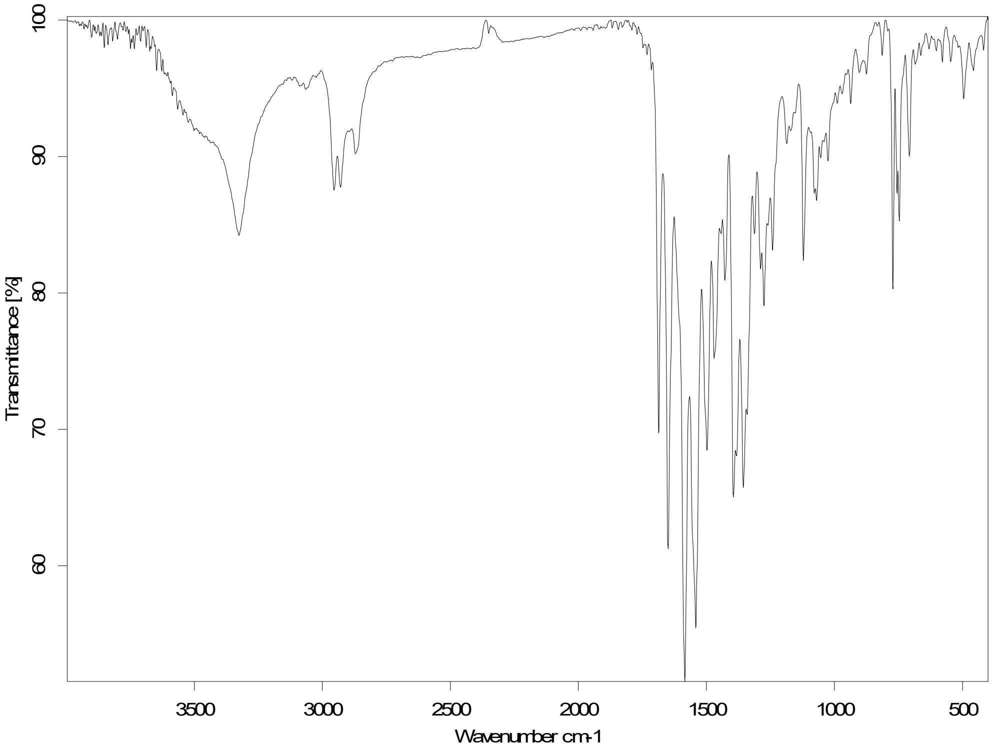

Supplement: Supplementary file 1 [file molecules-22-01741-s001.pdf]
